# Novel concepts of antiangiogenic therapies in metastatic renal cell cancer

**DOI:** 10.1007/s12254-017-0344-2

**Published:** 2017-08-11

**Authors:** Renate Pichler, Isabel Heidegger

**Affiliations:** Department of Urology, Medical University Innsbruck, Anichstraße 35, 6020 Innsbruck, Austria

**Keywords:** Renal cell carcinoma, Antiangiogenic therapy, Programmed-death ligand 1, Hypoxia, Resistance mechanism

## Abstract

The era of antiangiogenic drugs targeting the vascular endothelial growth factor (VEGF) signaling pathway has become a mainstay in the treatment of metastatic renal cell carcinoma (mRCC), showing primary responses in 65–70% of patients. Nevertheless, most of those patients progress to angiogenesis inhibitors over time due to different modes of resistance (adaptive and intrinsic). Both in vitro and in vivo analyses provided evidence that PD-L1 upregulation in hypoxia conditions is dependent on hypoxia-inducible factor (HIF)-2alpha and is associated with an overexpression of VEGF. Thus, additional blockade of PD-L1 along with inhibition of angiogenesis pathways seems to represent a novel and innovative treatment concept in mRCC. In this short review, we provide an overview on ongoing phase III trials combining antiangiogenic therapies with checkpoint inhibitors in the first-line setting. Moreover, we critically analyze the impact of recently approved therapeutic antiangiogenic agents and checkpoint inhibitors after progression to first-generation tyrosine kinase inhibitors and their mode of action. In addition, response and resistance hypotheses and biomarkers to antiangiogenic therapy in clinical practice are critically discussed.

Renal cell carcinoma (RCC) is the ninth most common cancer worldwide, with approximately 63,990 estimated new cases in 2017 in the United States [1]. Approximately 20–30% of patients present in a metastatic stage at the time of diagnosis. Approximately one third of those patients with initial curative surgical approach will develop local recurrence or distant metastases over time [2, 3]. Clear cell renal cell carcinoma (ccRCC), a subtype of RCC, is a highly vascularized tumor and is therefore an attractive disease to study angiogenesis and to test novel angiogenesis inhibitors in early clinical development. The introduction of vascular endothelial growth factor (VEGF)-targeted tyrosine kinase inhibitors (TKIs) 10 years ago has revolutionized the systemic treatment of metastatic RCC (mRCC) after the cytokine decade using interleukin-2 (IL-2) and interferon-alpha (IFN-alpha) [4]. Nevertheless, complete response is confirmed in less than 1% as most patients with initial response progress during antiangiogenic therapy due to diverse resistance mechanisms [2, 4].

## Biology of resistance and response hypotheses to antiangiogenic agents

**Fig. 1:**
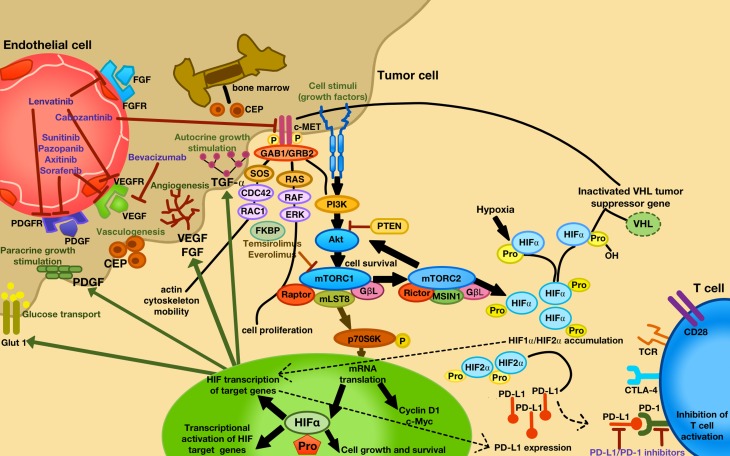
Approved VEGF-targeted and mTOR-targeted antiangiogenic drugs and their specific targets with their mode of action. Increased tumor hypoxia during antiangiogenic therapy is the key player for developing TKI resistance, with an accumulation of HIF-alpha. Consequently, different alternative HIF and/or non HIF-derived proangiogenic (e. g. ephrin, angiopoietin, FGF, VEGF, PIGF) and c‑MET (cell motility, proliferation, differentiation, migration and invasion) signaling pathways are activated, being responsible for further tumor progression. Moreover, hypoxia leads to an activation of bone marrow-derived cells consisting of circulating endothelial progenitor cell (CEP), forming new blood vessels in the tumor (vasculogenesis). Under hypoxia, PD-L1 upregulation was dependent on HIF-2a in RCC, being associated with simultaneous VEGF overexpression. *CEP* circulating endothelial progenitor, *FGF (R)* fibroblast growth factor (receptor), *PDGF (R)* platelet-derived growth factor (receptor), *PIGF* placental growth factor, *VEGF (R)* vascular endothelial growth factor (receptor)

In general, two different modes of resistance to antiangiogenic agents, the adaptive (evasive) and the intrinsic (pre-existing) non-responsiveness, have been described in preclinical models [5, 6]. The adaptive resistance is built on the concept that angiogenic tumors can develop an adaptation to VEGF-targeted therapy by evading the therapeutic blockade of angiogenesis due to an upregulation of alternative angiogenic and invasive pathways, including MET and AXL (receptor tyrosine kinases) [4, 5]. For example, it is well known that a chronic sunitinib therapy in RCC cell lines can induce MET and AXL signaling, thus promoting the epithelial-mesenchymal transition (EMT), with increased cell invasion, migration and angiogenesis [7]. Moreover, Von-Hippel-Lindau (VHL) mutations in ccRCC patients with antiangiogenic therapies induce a hypoxia-inducible factor (HIF)alpha accumulation, also activating alternative HIF and/or non HIF-mediated proangiogenic signaling pathways in the tumor, such as fibroblast growth factor (FGF), placental growth factor (PIGF), ephrin and angiopoietin [5]. In addition, a recruitment of bone marrow-derived cells consisting of vascular progenitors and proangiogenic monocytic cells for vasculogenesis is induced, limiting the obligatory necessity of VEGF signaling [8]. Another consequence of upregulated alternative angiogenic pathways is an increase of pericyte coverage for protecting tumor blood vessels [5] and tumor cell invasiveness to escape oxygen deprivation [5]. Moreover, a compensatory increase of the phosphatidylinositide 3‑kinase (PI3K) and protein kinase B (Akt/PKB) pathway due to mammalian target of rapamycin complex (mTORC)1 inhibition (everolimus, temsirolimus) may lead to an upregulation of mTORC2 with further Akt und HIF activation; however, whether mTOR inhibitors that target both mTOR complexes increase antitumor effects has yet to be tested in RCC ([9]; Fig. 1).

The intrinsic resistance to antiangiogenic therapies is explained by the fact that certain tumors have a pre-existing resistance, meaning that tumors have already activated evasive resistance mechanisms, before starting antiangiogenic therapy in response to the selective mechanisms within the microenvironment during pre-malignant transformation [5].

A major challenge in clinical practice, is to elucidate potential predictive biomarkers identifying those patients who mostly benefit from a certain antiangiogenic agents [10]. It has been previously reported that response or resistance to antiangiogenic agents may be evaluated by endothelial cell effects, such as therapy-induced hypertension [11], treatment-induced functional radiographic changes in tumor blood flow by dynamic contrast-enhanced magnetic resonance imaging (DCE-MRI) [10, 12] or by measuring levels of circulating endothelial cells (CEC), circulating endothelial progenitor cells (CEP) and tumor endothelial markers [10]. Another approach to overcome angiogenic escape may be a rechallenge of antiangiogenic drugs due to inadequate target inhibition based on increased receptor signaling and/or reduced drug levels [9]. Nevertheless, only few trials tested a comprehensive biomarker panel of possible resistance/response mechanisms during antiangiogenic therapy in the clinical setting [10, 13]. Thus, further validation of these preliminary results is obligatory before drawing any final conclusions.

## Tumor hypoxia and PD-L1 expression: a novel therapeutic approach in the first-line setting in mRCC?

The VEGF-targeted antiangiogenic agents induce tumor hypoxia, leading to an upregulation of programmed death-ligand 1 (PD-L1) in different cancer entities including hepatocellular carcinoma [14], lung cancer [15] and RCC [16]. Generally, hypoxic zones in the tumor can attract different immunosuppressive myeloid cells, such as myeloid-derived suppressor cells (MDSC). Under hypoxic conditions, HIF-1alpha leads to an upregulation of PD-L1 expression on MDSCs in the tumor microenvironment, thereby increasing interleukin (IL)-6 and IL-10 secretion from MDSCs, causing a MDSC-induced immunosuppression, T cell inactivation, and promoting tumor progression [17, 18]. In RCC, hypoxia, and in addition, a loss of the VHL protein (pVHL) results in the constitutive stabilization of HIF1alpha and HIF2alpha, inducing various HIF-transcriptional targets [19]. The PD-L1 as a HIF2alpha target was upregulated in VHL protein deficient ccRCC in vitro [20]. In RCC patients, PD-L1 expression positively correlated with VHL mutation, HIF-2alpha expression, adverse pathological features such as higher nuclear grade, necrosis and sarcomatoid transformation, c‑MET and VEGF expression [16, 21], thus resulting in a shorter progression-free and cancer-specific survival [21]. Based on these data, simultaneous blockade of PD-L1 with the inhibition of the VHL/HIF/VEGF pathway may represent a novel and innovative treatment concept [17]. Thus, various randomized phase III trials in the first-line setting of mRCC are currently ongoing, testing this combined therapeutic approach consisting of checkpoint inhibitors (avelumab, pembrolizumab, atezolizumab, nivolumab, ipilimumab) combined with VEGF-targeted antiangiogenic agents (axitinib, lenvatinib, bevacizumab) in comparison to standard first-line drugs alone (sunitinib) (Table 1). Results of these trials are expected soon.

**Table 1:** Ongoing phase III trials in the first-line setting of mRCC, focusing on the combination of VEGF-targeted antiangiogenic drugs and checkpoint inhibitors

Agents	Targets	Comparator	Study	Study phase	Status	Estimated patient enrollment	Study registration number	Primary outcome measures	Secondary outcome measures
Avelumab + Axitinib	PD-L1 VEGFR-1/2/3	Sunitinib	JAVELIN RENAL 101	III	Recruiting	583	NCT02684006	PFS	OS OR DCR DOR TTR EQ-5D/FKSI-19
Lenvatinib + Everolimus or	VEGFR-1/2/3 FGFR-1/2/3/4	Sunitinib	E7080-G000-307	III	Recruiting	735	NCT02811861	PFS	ORR OS HRQoL PFS2
Lenvatinib + Pembrolizumab	PDGFR-alpha RET c-KIT mTOR PD-1								
Pembrolizumab + Axitinib	PD-1 VEGFR-1/2/3	Sunitinib	KEYNOTE-426	III	Recruiting	840	NCT02853331	PFS OS	ORR DCR AEs
Atezolizumab + Bevacizumab	PD-L1 VEGF	Sunitinib	ImMotion151	III	Ongoing, but not recruiting	915	NCT02420821	PFS OS	OS (PD-L1) CR/PR (%) DOR
Nivolumab + Ipilimumab	PD-1 CTLA-4	Sunitinib	CheckMate-214	III	Ongoing, not recruiting	1070	NCT02231749	PFS OS ORR	AE rate

*AE* adverse events, *CR* complete response, *DCR* disease control rate, *DOR* duration of response, *EQ-5D* EuroQuality of life, *FKSI-19* Functional Assessment of Cancer Therapy-Kidney Symptom Index, *HRQoL* health-related quality of life, *ORR* objective response rate, *OR* objective response, *OS* overall survival, *PDGF (R)* platelet-derived growth factor (receptor), *PFS* progression-free survival, *PR* partial response, *TTR* time to tumor response

## Novel approved TKIs and checkpoint inhibitors in mRCC after progression to first-generation VEGF-targeted antiangiogenic agents

**Fig. 2:**
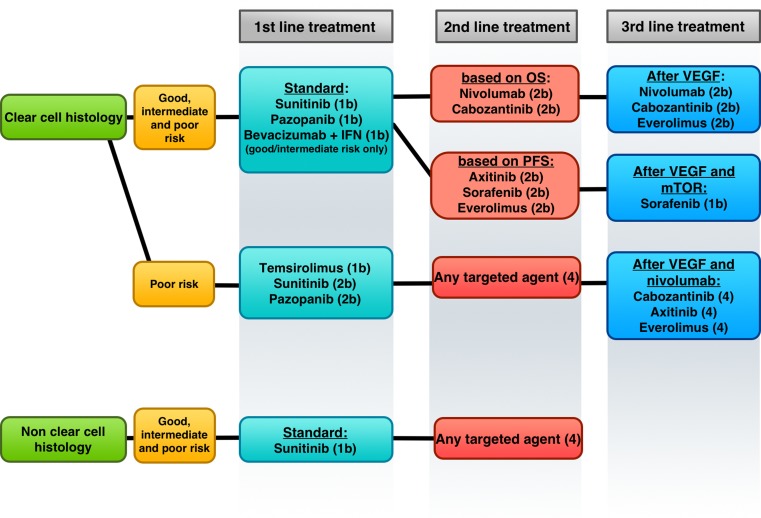
Schematic overview of the current European Association of Urology 2017 guidelines and evidence-based recommendations for systemic treatment in mRCC (level of evidence). *OS *overall survival, *PFS* progression-free survival, *VEGF* vascular endothelial growth factor, *mTOR* mammalian target of rapamycin. (Adapted from [22])

In 2016, the Food and Drug Administration (FDA) and the European Medicines Agency (EMA) have approved to two novel TKIs, cabozantinib and lenvatinib (in combination with the mTOR inhibitor everolimus) and one PD-1 inhibitor, nivolumab, after progression to first-generation VEGF-targeted TKIs in mRCC, changing and radically improving the sequence therapy in the second-line, third-line and in the later line setting. A schematic overview of the current European Association of Urology (EAU) 2017 guidelines with evidence-based recommendations for systemic treatment in mRCC is shown in Fig. 2.

### Cabozantinib

This is an oral multi-TKI blocking VEGFR-1, 2, 3, RET, KIT, TRKB, FLT-3, AXL, TIE-2, with the additional potential to inhibit c‑MET. The c‑MET expression was noticed to be an independent prognostic marker and a potential therapeutic target especially in ccRCC, associated with worse disease-specific survival [23] due to aggressive tumor behavior [24] and increased PD-L1 expression [21]. The open-label, randomized phase III METEOR trial included 658 mRCC patients with previous treatment with one or more VEGF-targeted TKIs, receiving cabozantinib 60 mg or everolimus 10 mg once per day consecutively. Interestingly, the median progression-free survival (PFS) (cab vs. eve: 7.4 vs. 3.9 months; hazard ratio HR = 0.51; *p* < 0.0001) as well as the overall survival (OS) (cab vs. eve: 21.4 vs.16.5 months; HR = 0.66; *p* = 0.00026) was significantly increased in the cabozantinib group compared to everolimus, thus becoming one of the new efficient second-line TKIs in the recent European Society of Medical Oncology (ESMO) and EAU guidelines. Nevertheless, a dose reduction during treatment occurred in 60% of all patients undergoing cabozantinib therapy, with serious adverse events grades 3/4 in 39% [25].

### Lenvatinib

This is a multi-target TKI of VEGFR-1, -2, -3 also inhibiting FGFR-1, -2, -3 and -4, PDGFRalpha, KIT and RET. Antiangiogenesis activity and antitumor cell growth of lenvatinib was previously confirmed by inhibiting VEGF and FGF-driven proliferation and tube formation of human umbilical vein endothelial cells in vitro. In addition, in vivo angiogenesis induced by overexpressed VEGF or FGF was significantly suppressed with oral lenvatinib treatment [26]. The enhanced antitumor activity by combining lenvatinib plus everolimus may be explained by the simultaneous targeting of tumor cell growth and angiogenesis in human RCC xenograft models [27]. This combination confirmed an additive activity in VEGF-activated, and synergistic activity against FGF-activated endothelial cells, with suppression of mTOR-S6K-S6 signaling [27]. In the clinical setting, the phase 1b study confirmed lenvatinib 18 mg and everolimus 5 mg once a day as the maximum tolerated dose in patients with mRCC, with manageable toxicity and the best therapeutic response (stable disease in 45.5% and partial remission in 36.4%) [28]. The following phase II trial with 153 patients who progressed after first-line VEGF-targeted therapy received either lenvatinib 18 mg combined with everolimus 5 mg, single-agent lenvatinib 24 mg, or single-agent everolimus 10 mg. Compared to lenvatinib and everolimus monotherapy, the combination of lenvatinib and everolimus showed the best median PFS (14.6 months) and median OS (25.5 months), with diarrhea as the most common grade 3/4 adverse event in 20% [29].

Based on the limited size of approximately 150 patients in this phase II study, the combination of lenvatinib and everolimus was not, at this stage, recommended either by current ESMO 2016 [30] or by EAU 2017 guidelines [22] on RCC as a novel second-line therapeutic regimen.

### Nivolumab

This is the first approved PD-1 checkpoint inhibitor in the second-line treatment of mRCC. The randomized phase II trial evaluated three doses of nivolumab (0.3, 2 and 10 mg/kg intravenously once every 3 weeks) to identify a potential dose-response relationship and assess the activity and safety of nivolumab in patients with mRCC. Interestingly, no dose-dependent relationship was confirmed by PFS (2.7 vs. 4.0 vs. 4.2 months, respectively) and ORR (20% vs. 22% vs. 20%, respectively) with manageable safety profiles across the three doses (grade 3–4 adverse events AE: 5% vs. 17% vs. 13%, respectively) [31]. The following phase III Checkmate 025 trial compared nivolumab (3 mg/kg intravenously every 2 weeks) with everolimus (10 mg orally once a day) in patients who received previous treatment with one or two regimens of antiangiogenic therapy. Nivolumab confirmed significantly better median OS (25.0 vs. 19.6 months) and ORR (25% vs. 5%, OR = 5.98) in comparison to everolimus [32]. In a further subgroup OS analyses of the Checkmate 025 study population, nivolumab confirmed an OS improvement versus everolimus across all subgroups including age, number of sites of metastases, type of metastases, number and duration of prior therapies, type of prior therapy, and Memorial Sloan Kettering Cancer Center (MSKCC) risk groups, with a high benefit in patients belonging to the poor MSKCC group [33]. Moreover, the rate of grade 3 or 4 AEs was less in patients treated with nivolumab (19%) compared to everolimus (37%) [32], thus resulting in a significant improvement of health-related quality of life in patients treated with nivolumab versus everolimus (55% vs. 37%, *p* < 0.0001) [34].

## Conclusion

Angiogenic tumors can develop an adaptation to VEGF-targeted therapy by evading the therapeutic blockade of angiogenesis due to an upregulation of alternative angiogenic and invasive pathways. The VEGF-targeted antiangiogenic-induced tumor hypoxia leads to an upregulation of HIF1 and 2alpha, thus activating survival pathways in the tumor cells with an increased activation of proangiogenic signaling pathways. In addition, PD-L1 expression is upregulated by HIF-2alpha in RCC. Thus, the combined therapeutic approach simultaneously inhibiting the VHL/HIF/VEGF pathway and the PD-L1 expression seems to be an attractive and efficient method for increasing antitumor activity in mRCC. Several phase III clinical trials are currently investigating the combination of TKIs plus immunotherapy compared to TKI alone in the first-line setting, the results of which are expected soon.
